# Postpartum spontaneous dissection of the first obtuse marginal branch of the left circumflex coronary artery causing acute coronary syndrome: a case report and literature review

**DOI:** 10.1186/1752-1947-7-82

**Published:** 2013-03-19

**Authors:** Khurram Shahzad, Long Cao, Quara Tul Ain, Jennifer Waddy, Nawazish Khan, Rajasekhar Nekkanti

**Affiliations:** 1 Department of Internal Medicine, Division of Cardiology, East Carolina University, Brody School of Medicine, 600 Moye Boulevard, Greenville NC 27834, USA

## Abstract

**Introduction:**

Spontaneous coronary artery dissection is a rare but important cause of acute coronary syndrome. It can cause unstable angina, acute myocardial infarction, and sudden death. The condition commonly affects young females with about one-third of the cases occurring during pregnancy and the peripartum period. The diagnosis may occasionally be overlooked as the patients are often young and have no risk factors for coronary artery disease.

**Case presentation:**

Here we report the case of a 29-year-old African American woman who presented with acute coronary syndrome due to spontaneous dissection of the first obtuse marginal branch of the left circumflex coronary artery at three weeks post-partum and recovered requiring only medical management, possibly by longitudinal distribution of the intramural hematoma leading to good distal flow.

**Conclusions:**

Spontaneous coronary artery dissection should be suspected in all young multiparous females presenting with chest pain in the peripartum period even in the absence of risk factors. Urgent diagnosis by angiography is required. It is recommended that treatment should be tailored to meet individual circumstances. Patients who present with single-vessel disease and hemodynamic stability, and who receive medical treatment with anticoagulation, nitrates and a beta-blocker, should experience good results.

## Introduction

Spontaneous coronary artery dissection (SCAD) is a rare cause of acute coronary syndrome with less understood pathophysiological mechanisms. Coronary artery dissection can occur spontaneously or as a consequence of chest trauma, cardiac surgery, coronary angiography, coronary intervention, or as an extension of aortic dissection. SCAD was first described in 1931, during autopsy findings in a 42-year-old woman who died after presenting with chest pain [1]. Approximately 400 documented cases of SCAD have been reported. This is likely an underestimate due to a significant number of spontaneous dissections presenting with sudden death [2,3]. The overall incidence of SCAD in angiographic series ranges from 0.28% to 1.1% [4,5]. There is a predominance of SCAD in young women (70%) of which approximately 30% of cases occur in the peripartum period [6]. The true etiopathological mechanisms remain unclear. Possible risk factors of the cases occurring in the peripartum period include hormonal changes, hemodynamic stress and changes in autoimmune status.

The left anterior descending (LAD) artery is the most frequent location of dissection. In angiographic and autopsy series, the LAD accounts for over 60% of coronary dissections [6,7]. Involvement of the right coronary artery (RCA), left main coronary artery, and left circumflex (LCX) artery is uncommon.

The diagnosis of SCAD may occasionally be overlooked as the patients are often young and have no risk factors for coronary artery disease. Historically, most cases have been diagnosed on autopsy until the first angiographic diagnosis in 1978 [8]. Because of the rarity of the condition, no management guidelines exist. Here we describe the clinical presentation and interesting angiographic findings in a case of acute coronary syndrome with dissection of the first obtuse marginal (OM1) branch of the LCX with a brief discussion of a possible mechanism of recovery in the context of current management options.

## Case presentation

A 29-year-old African American woman was admitted with sudden onset of substernal chest pain. She was three weeks post-partum and this was her fifth baby. Her pregnancy and delivery was uneventful. Pain started while she was feeding her newborn. It was sharp, substernal, associated with shortness of breath (SOB) and diaphoresis. She also noticed left arm numbness and tingling. She had no past medical history of chest pain, hypertension, diabetes mellitus, connective tissue disease or heart failure. There was no family history of premature coronary artery disease (CAD). On admission, her electrocardiogram showed bigeminy without any ST-segment or T-wave changes. Cardiac enzymes were elevated with the peak troponin level of 31ng/ml. Based on this presentation, diagnosis of non-ST-segment elevation myocardial infarction (NSTEMI) was made and the patient was started on heparin, clopidogrel (Plavix), beta-blocker, and nitrates. To rule out pulmonary embolism (PE) the patient underwent a computerized tomographic angiogram (CTA) of her chest, which was found to be normal. The patient was transferred to our facility for further management.

**Figure 1:**
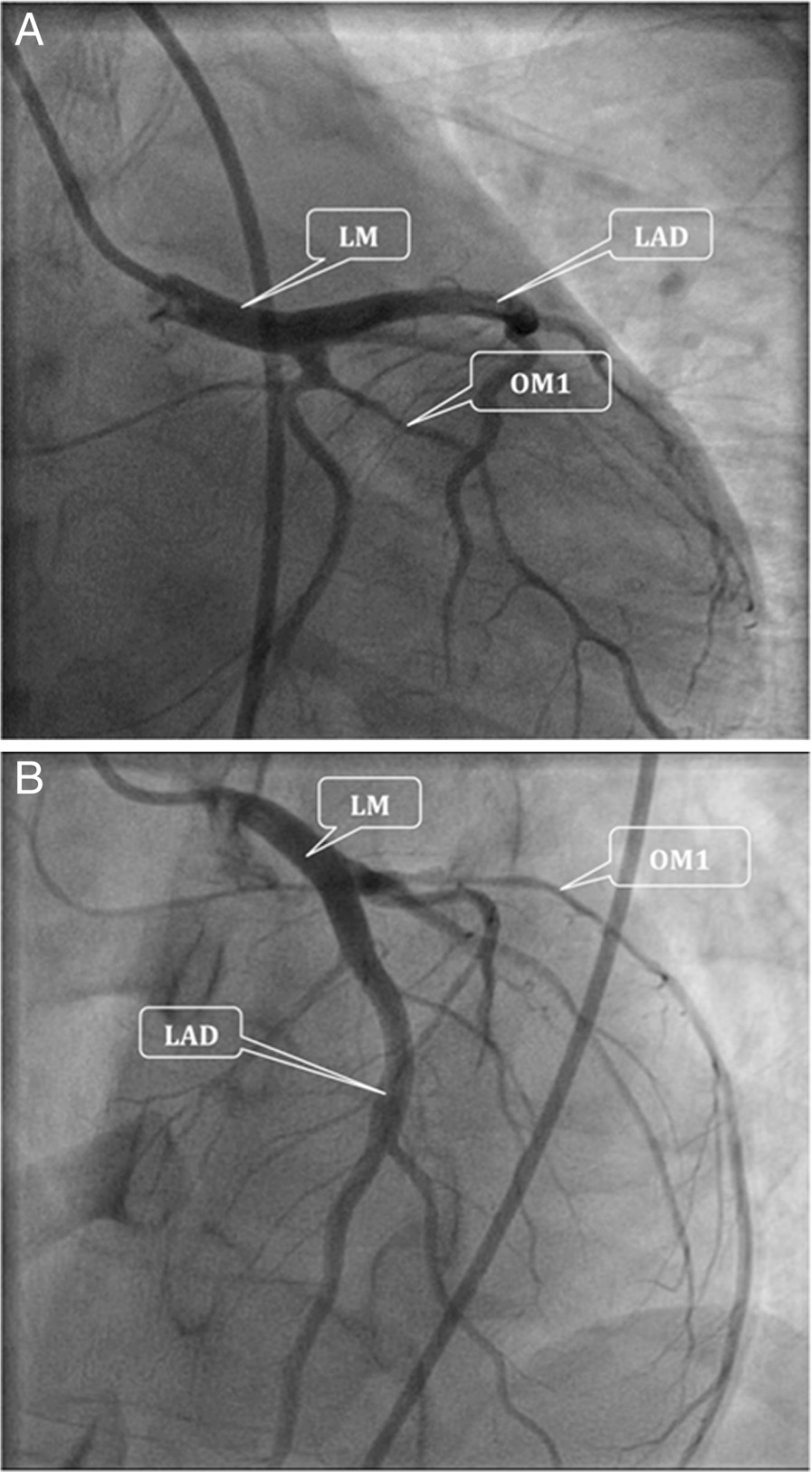
Coronary angiograms showing the obtuse marginal branch with good distal flow in lateral view (A) and anterior view (B).

She underwent left heart catheterization (LHC) that revealed dissection of the first obtuse marginal branch (OM1) of the left circumflex artery extending from the proximal to mid-distal end. At the very distal end of the vessel, the lumen was of normal diameter with good distal flow (Figure 1). Left main artery, LAD including its all three diagonal branches and RCA were without any significant disease. To assess the cardiac function left ventriculography was performed that showed left ventricular global hypokinesis with an ejection fraction (EF) of 30% to 35%. There were no regional wall motion and valvular abnormalities. The diseased area of the involved vessel was too long for stenting so the decision was made to manage her conservatively with aspirin, clopidogrel (Plavix) and a beta- blocker. She remained stable throughout her hospital stay and recovered without any symptoms. She was discharged home on day three of admission with clopidogrel (Plavix), nitrates and a beta-blocker with close outpatient follow-up.

## Discussion

Peripartum dissection has been associated with the presence of eosinophils. Eosinophilic infiltrates have been described in the coronary artery adventitia in autopsy studies of SCAD without coronary atherosclerosis [9]. Eosinophilic granules contain numerous lytic substances, including collagenase, peroxidase, major basic protein, and acid phosphatase. These substances may break down the medial-adventitial layers and lead to dissection. During labor and the peripartum period, eosinophils infiltrate the uterus and serum collagenase levels increase. The presence of eosinophils in dissected coronary arteries may be a systemic manifestation of this process [10]. In animal studies, estrogen and progesterone can induce eosinophils to release granules that contain lytic substances [11,12]. Elevations of estrogen and progesterone occur during pregnancy and the peripartum period. In addition, during pregnancy there are microstructural changes in the elastic and collagen fibers of the tunica media of the aorta that may be caused by hormonal and hemodynamic factors [13]. These changes could also occur in the coronary arteries, possibly contributing to the predisposition to dissection in the peripartum period [14].

**Figure 2:**
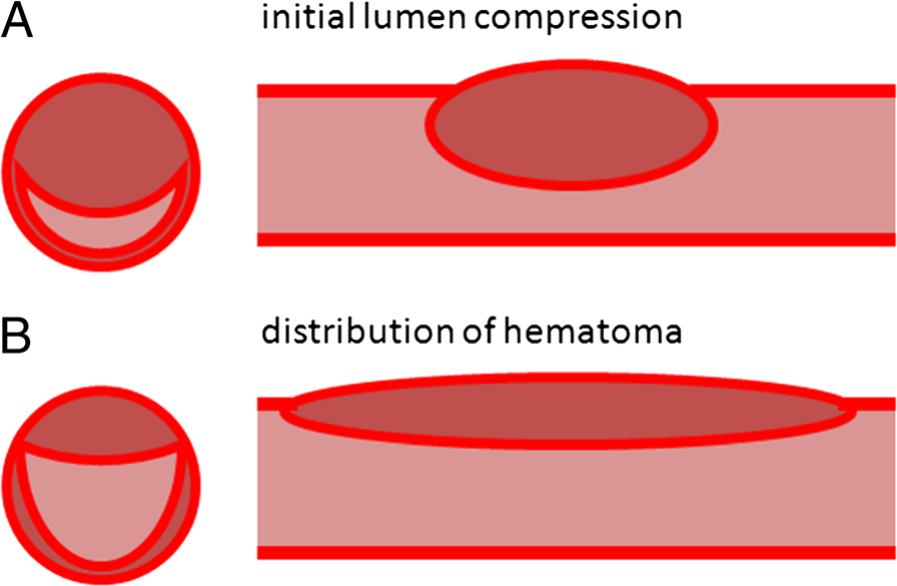
Hypothetical representation of the formation of intramural hematoma caused by coronary artery dissection leading to initial lumen compression and acute coronary syndrome (A) and later longitudinal distribution of hematoma caused by mechanical pressure leading to development of good distal blood flow with resolution of symptoms (B).

Coronary artery dissection has been reported in relationship to artery spasm for example caused by cocaine use [15] and variant angina [16]. Inflammatory mechanisms of eosinophils described in the peripartum period may play a role in spasm as well. In an animal model, extracts from eosinophils caused strong contraction of intestinal smooth muscle [17]. It is possible that eosinophilic products can trigger coronary spasm in humans. Preexisting connective tissue diseases such as Marfan syndrome and Ehlers-Danlos type IV and systemic lupus erythematosus (SLE)-mediated vasculitis can also predispose to spontaneous dissections. Dissection of the coronary artery results in separation of the layers of the arterial wall, creating a false lumen. The separation may be between the intima and the media, or between the media and the adventitia. Hemorrhage into the false lumen can impinge upon the true lumen of the coronary artery, impairing blood flow and causing myocardial ischemia, unstable angina, infarction, or sudden death (Figure 2) [18-20].

The location and distribution of dissection in our case is very important. The involvement of the OM1 branch, as in our case, had been rarely reported in literature. The longitudinal distribution of dissection from proximal to mid-distal end of the OM1 with normal distal flow can only explain the early recovery process but not the initial presentation of the patient. We hypothesize that maybe in our patient there was a development of hematoma coexisting with the arterial spasm or later development of spasm. The sheer pressure generated by the spasm, with the preexisting separation of wall layers, might have caused the longitudinal distribution of the hematoma with the decrease in the lumen blockage and improvement in the distal flow (Figure 2). This longitudinal distribution luckily not only saved the patient from undergoing coronary intervention, but also helped recovery with only medical management.

Management of SCAD depends on the clinical presentation and individual circumstances including site of the dissection, the number of vessels involved and the coronary flow status [21]. Hemodynamic status, often related to the extent of the dissection, also determines management. Medical treatment and percutaneous coronary intervention (PCI) are usually sufficient to restore coronary flow and stable hemodynamics [22]. Surgery with coronary artery bypass grafting (CABG) is considered in severe cases with multiple vessel disease, presentation with hemodynamic instability and/or in cases with failure of revascularization. In a series of 23 cases, Vanzetto *et al*. [23] reported that bypass was performed five times for four left main coronary dissections and one LAD coronary dissection and angioplasty eight times for proximal large trunk lesions, while ten distal dissections were managed medically.

Before the angiographic diagnosis during the acute phase, some patients are managed by thrombolysis. Thrombolytics have been accused of favoring intramedial hematoma extension and compression of the true lumen. They could indeed be harmful. Glycoprotein IIb/IIIa inhibitors are likewise contraindicated. Once the diagnosis of SCAD has been confirmed, in the case of stable hemodynamics and single-vessel disease affecting a limited territory, medical treatment is a reasonable option as was the case with our patient. Medical treatment involves anticoagulation with heparin for the first days, given the risk of thrombosis of the true channel due to slowed flow, antiplatelet therapy and beta-blockers. The combination of acetylsalicylic acid and clopidogrel may be preferable to monotherapy [24].

In case of medical management, close surveillance is necessary with angiographic control for the first three months. Evolution may be toward spontaneous cicatrization (in 50% of cases), persistent dissection with or without ischemia [25] or formation of a pseudoaneurysm [26].

## Conclusions

SCAD is a rare but serious cause of acute coronary syndrome with the majority of cases associated with pregnancy. SCAD should be suspected in all young multiparous women presenting with chest pain in the peripartum period, even in the absence of risk factors. Urgent diagnosis by angiography is required. It is recommended that treatment should be tailored to meet individual circumstances. Presentation with single-vessel disease and hemodynamic stability, medical treatment with anticoagulation, nitrates and a beta-blocker should produce good results.

## Consent

Written informed consent was obtained from the patient for publication of this case report and accompanying images. A copy of the written consent is available for review by the Editor-in-Chief of this journal.

## Abbreviations

CABG: Coronary artery bypass grafting; CAD: Coronary artery disease; CTA: Computerized tomographic angiogram; EF: Ejection fraction; LAD: Left anterior descending; LCX: Left circumflex; LHC: Left heart catheterization; NSTEMI: Non-ST-segment elevation myocardial infarction; OM1: First obtuse marginal branch; PCI: Percutaneous coronary intervention; PE: Pulmonary embolism; RCA: Right coronary artery; SCAD: Spontaneous coronary artery dissection; SLE: Systemic lupus erythematosus; SOB: Shortness of breath.

## Competing interests

The authors declare that they have no competing interests.

## Authors’ contributions

KS prepared the initial manuscript and performed all the editing leading to its current form. LC was involved in the clinical care of the patient. QTA analyzed and interpreted the patient data regarding the clinical presentation of the disease. JW was involved in the clinical care of the patients. NK performed the radiological examination of the coronary arteries and helped in writing the manuscript. RN helped analyze the presentation of the manuscript and oversaw the development to its current form. All authors read and approved the final manuscript.
